# Evaluation of co-circulating pathogens and microbiome from COVID-19 infections

**DOI:** 10.1371/journal.pone.0278543

**Published:** 2022-12-01

**Authors:** James B. Thissen, Michael D. Morrison, Nisha Mulakken, William C. Nelson, Chris Daum, Sharon Messenger, Debra A. Wadford, Crystal Jaing

**Affiliations:** 1 Physical and Life Sciences Directorate, Lawrence Livermore National Laboratory, Livermore, CA, United States of America; 2 Computating Directorate, Lawrence Livermore National Laboratory, Livermore, CA, United States of America; 3 Earth and Biological Sciences Directorate, Pacific Northwest National Laboratory, Richland, WA, United States of America; 4 Joint Genome Institute, Berkeley, CA, United States of America; 5 Viral and Rickettsial Disease Laboratory, California Department of Public Health, Richmond, CA, United States of America; Nigerian Institute of Medical Research, NIGERIA

## Abstract

Co-infections or secondary infections with SARS-CoV-2 have the potential to affect disease severity and morbidity. Additionally, the potential influence of the nasal microbiome on COVID-19 illness is not well understood. In this study, we analyzed 203 residual samples, originally submitted for SARS-CoV-2 testing, for the presence of viral, bacterial, and fungal pathogens and non-pathogens using a comprehensive microarray technology, the Lawrence Livermore Microbial Detection Array (LLMDA). Eighty-seven percent of the samples were nasopharyngeal samples, and 23% of the samples were oral, nasal and oral pharyngeal swabs. We conducted bioinformatics analyses to examine differences in microbial populations of these samples, as a proxy for the nasal and oral microbiome, from SARS-CoV-2 positive and negative specimens. We found 91% concordance with the LLMDA relative to a diagnostic RT-qPCR assay for detection of SARS-CoV-2. Sixteen percent of all the samples (32/203) revealed the presence of an opportunistic bacterial or frank viral pathogen with the potential to cause co-infections. The two most detected bacteria, *Streptococcus pyogenes* and *Streptococcus pneumoniae*, were present in both SARS-CoV-2 positive and negative samples. Human metapneumovirus was the most prevalent viral pathogen in the SARS-CoV-2 negative samples. Sequence analysis of 16S rRNA was also conducted to evaluate bacterial diversity and confirm LLMDA results.

## Introduction

The emergence of SARS-CoV-2 in late 2019 has severely impacted global health, lives, and livelihoods over the last 2 years. During the first months of the COVID-19 pandemic, beyond singular infection with SARS-CoV-2, reports of co-infection with other respiratory pathogens emerged [1–3]. In the study by Chen et al, 51% of the 99 patients with SARS-CoV-2 from Wuhan China in January 2020 had comorbid conditions [1]. Kim et al analyzed more than 1,200 nasopharyngeal swabs collected from Northern California in March 2020 and found that 26% of the samples were positive for one or more co-infecting pathogens [2]. A meta-analysis of 118 studies published between October 1, 2019 and February 8, 2021 showed as many as 19% of patients with COVID-19 had co-infections [3]. The three most frequently identified viruses among SARS-CoV-2 samples from this meta-analysis were influenza type A, influenza type B, and respiratory syncytial virus (RSV), while the three most frequently identified bacteria were *Klebsiella pneumoniae*, *Streptococcus pneumoniae*, and *Staphylococcus aureus*; *Aspergillus* spp. were the most frequently reported fungi among those with co-infections [3]. The presence of bacterial co-infection was associated with poor outcomes, including increased mortality [4]. Additionally, a multi-center study of 905 patients from January to February 2020 reported clinically diagnosed bacterial co-infections from 9.5% of COVID-19 patients [5]. These studies have used real-time PCR to determine the presence of co-infecting pathogens. Real-time PCR offers sensitive detection but limits the breadth of detection to known or suspected targets for which PCR assays are available. The current study describes the use of a more comprehensive and multiplexed detection platform that enables simultaneous detection of multiple viral, bacterial and fungal infections, even those not suspected initially.

In this report, we aimed to evaluate both the burden of co-infections in patients with COVID-19, as well as evidence for differences in microbiome between SARS-CoV-2 positive vs SARS-CoV-2 negative samples. We analyzed a total of 203 residual clinical samples, 101 SARS-CoV-2 positive and 102 SARS-CoV-2 negative samples, originally submitted to the California Department of Public Health (CDPH) for SARS-CoV-2 diagnostic testing between February 2020 and July 2020. In contrast to some earlier studies where samples were collected before February 2020, when other viral pathogens were known to be circulating [2, 4], the majority of samples in this study were collected after the declaration of a statewide Shelter-in-Place on March 19, 2020, co-incident with a decline in circulating respiratory viral pathogens.

We used the Lawrence Livermore Microbial Detection Array (LLMDA) to analyze these samples. The LLMDA is a broad-spectrum microbial detection platform which contains DNA probes to detect more than 12,000 microbial species including viruses, bacteria, fungi, protozoa and archaea. The LLMDA has been applied to a variety of human and animal clinical samples to identify pathogens in disease cases and assess the microbiome differences between healthy and diseased samples [6–11]. The LLMDA (v7) has been applied to veterinary diagnostics and surveillance of viral diseases in the field [7, 12, 13]. The latest version of the LLMDA was the Applied Biosystems Axiom Microbiome Array that can process 24 or 96 samples simultaneously [14].

Sequence analysis of 16S rRNA was conducted as a complementary method to evaluate the microbiome from the study samples. Bioinformatics and statistical analyses were conducted to evaluate the microbial profiles in the 203 samples.

## Methods

### Swab sample collection and SARS-CoV-2 PCR analysis

#### Clinical swab sample collection

Samples were provided by the California Department of Public Health/Viral and Rickettsial Disease Laboratory (CDPH/VRDL). There were no human subjects involved with this work and no consent was obtained or required. This work involved residual clinical diagnostic specimens. All samples were de-identified and analyzed anonymously. We obtained research exemption as deemed by the Committee for the Protection of Human Subjects (Project number 2020–127) issued under the California Health and Human Services Agency’s Federal Wide Assurance #00000681 with the Office of Human Research Protections. The work was done for public health surveillance purposes to better understand the pandemic. Samples were collected from individuals from various counties in the state of California from February 2020 to July 2020 for SARS-CoV-2 testing using a sample collection protocol described previously [15, 16]. A total of 203 samples were shipped to Lawrence Livermore National Laboratory (LLNL) for array analysis, of which, 102 were SARS-CoV-2 negative samples and 101 were SARS-CoV-2 positive samples, all tested at CDPH/VRDL. The list of the samples run on the LLMDA is shown in S1 Table. Of the 203 samples, 177 were nasopharyngeal (NP) swabs (87 SARS-CoV-2 positive, 90 SARS-CoV-2 negative) and 26 were nasal/oral pharyngeal/throat samples (14 SARS-CoV-2 positive, 12 SARS-CoV-2 negative). Six samples were oral pharyngeal (OP) swabs, of which all 6 were SARS-CoV-2 positive; 20 samples were nose/throat swabs, of which 8 were SARS-CoV-2 positive, and 12 were SARS-CoV-2 negative. Clinical information was only available for 53 samples (26%), 4 of which were from asymptomatic subjects. No clinical data were available for the other 148 (73%) samples.

#### SARS-CoV-2 PCR analysis

The CDPH/VRDL performed real-time reverse transcription-polymerase chain reaction (RT-qPCR) on the 203 samples described above for SARS-CoV-2. Prior to May 21, 2020 [15], samples were extracted using Qiagen DSP Viral RNA Mini Kit with carrier RNA added (Qiagen) with extracts tested for SARS-CoV-2 using the FDA EUA approved 2019-nCoV CDC Real-Time RT-PCR Diagnostic Panel assay, which targets two regions of the nucleoprotein gene (N1 and N2). After May 21, 2020 [16], samples were extracted using the KingFisher Flex (Thermo Fisher Scientific) instrument according to the manufacturer’s instructions. These later samples were tested using the FDA EUA approved the Taqpath™ Multiplex Real-time RT-PCR test, which includes nucleoprotein (N) gene, spike (S) gene, and ORF1ab gene targets.

### Microarray analysis of swab samples

#### Nucleic acid extraction

For this study, total nucleic acid was extracted from residual clinical swab samples in Viral Transport Media (VTM) using the MagMAX Microbiome Ultra Nucleic Acid Isolation Kit (Thermo Fisher Scientific). The nucleic acid in the extracted samples was quantified using a Qubit fluorimeter (Thermo Fisher Scientific). A range of DNA concentration was obtained, from 0.04 ng/μL to 156 ng/μL with an average concentration of 5.7 ng/μL. The RNA concentration ranged from non-detectable to 24.8 ng/μL.

#### Addition of SARS-CoV-2 probes to the LLMDA

In this study, the LLMDA v7 was used because it has the flexibility to update with SARS-CoV-2 probes. The v7 was developed in 2014 and can detect 4,219 viruses, 5,367 bacteria, 293 archaebacteria, 265 fungi, and 117 protozoa. All possible 60-mers from 41,540 SARS-CoV-2 genomes downloaded from GISAID in June 2020 were generated using Jellyfish 2.2.10 for evaluation as signatures. Only complete, medium or high coverage genomes from GISAID were included for this analysis. Any genome with over 3,000 N’s or genomic length below 28,000 nucleotides (nt) were filtered out. Only viruses isolated from human hosts were included. To find unique 60-mers, the 60-mers were mapped with BLAST against an “anti-target” sequence set consisting of all virus families other than Coronaviridae from NCBI and SARS-CoV-1, as well as the human genome. A hybridization probability score based on entropy, BLAST bit score, GC content, and number of mismatches was computed for every BLAST hit [12]. 60-mers with a probability of hybridization of over 20% to any anti-target genome was filtered out, leaving 365,292 unique k-mers.

The next step was to determine which of the unique k-mers were also highly conserved among the SARS-CoV-2 genomes. 42 BLAST databases were created out of the genomes to parallelize the conservation analysis. After the unique 60-mers were BLASTed to the target genomes, the same hybridization probability score was calculated for each BLAST result. This time 60-mers that had at least 95% probability of hybridizing to any of the target genomes were kept. High scoring 60-mers were split into several categories. First, 60-mers that map to almost all target genomes and those that are less conserved were separated since the less conserved 60-mers may be useful in distinguishing viral targets in different samples. Next, each of those two groups of 60-mers were split by genomic location to make it easier to select signature regions across the genome for assay design. Ensuring the final set of probes span the entire genome is important in protecting the ability to detect the virus in degraded samples.

#### Swab sample testing on the LLMDA

The updated LLMDA with SARS-CoV-2 probes was ordered from Agilent Technologies in the 4x180K format. The LLMDA analysis was carried out as described previously [7, 17]. Where possible, 10–20 ng of RNA was used as input into this protocol. Several samples did not have an RNA concentration that allowed for 10–20 ng of input and for these samples 8 μL was used as input. After array hybridization, washing and scanning, the fluorescent intensity data was extracted from the microarray images using the Feature Extraction Software (Agilent). The resulting intensity data was analyzed using the Composite Likelihood Maximization Method (CLiMax) [18]. The CLiMax analysis method requires that at least 20% of target-specific probes have a signal intensity above the 95^th^ or 99^th^ percentile of the control probes for a positive result. For the analysis of all the SARS-CoV-2 positive and negative samples, a threshold of 99% was used for detection. For the SARS-CoV-2 positive samples that were positive by PCR but negative by LLMDA at 99% threshold, a 95% threshold was also used to determine if SARS-CoV-2 can be detected at 95%.

Positive control standards were used to test the sensitivity of SARS-CoV-2 probes. The positive controls used included SARS-CoV-2 WA strain NR52285 (BEI), SARS-CoV-2 Italy strain NR52498 (BEI). Five μL of the extracted RNA was used for microarray analysis. A synthetic SARS-CoV-2 RNA Control 1 (MT007544.1) (Twist Bioscience) was used to spike into a negative CDPH sample at 10^6 and 10^5 copies.

#### LLMDA microbial detection prevalence analysis

The prevalence of species was calculated for both SARS-CoV-2 positive and negative samples. Significance testing of prevalence between the two groups was performed using the prop.test [19] function available in the stats package in R, and all P values were adjusted using the Benjamini-Hochberg method [20].

### 16S rRNA sequence analysis of swab samples

A total of 201 samples were run using 16S rRNA sequencing. Two of the samples (SARS-CoV-2 negative) that were run on LLMDA were not included in the 16S run due to low sample volume. Plate-based 16S V4 region sequencing library preps were performed on the Hamilton Vantage robotic liquid handling system using variable sample input up to a maximum of 30 ng, custom designed target primers with incorporated Illumina sequencing adapters, and the 5 PRIME HotMasterMix amplification kit with 30 cycles of PCR. Target primer sequences used for the 16S V4 region were 515F (GTGYCAGCMGCCGCGGTAA) and 806R (GGACTACNVGGGTWTCTAAT). After library sample preparation, the samples were pooled, and the pool quantified using KAPA Biosystem’s next-generation sequencing library qPCR kit and run on a Roche LightCycler 480 real-time PCR instrument. The pool was then loaded and sequenced on the Illumina MiSeq sequencing platform utilizing a MiSeq Reagent Kit, v3 600 cycle, following a 2x300 indexed run recipe. Reads were demultiplexed using Illumina’s bcl2fastq software. Raw fastq data was submitted to NCBI BioProject under accession RPJNA833483.

Fastq reads were imported into QIIME2 for analysis [21]. Sequences were truncated to 220 bp and the first 6 nt were trimmed off, as guided by the quality scores. Sequences were clustered to amplicon sequence variants (ASVs) using the dada2 algorithm [22], using min-fold-parent-over-abundance = 6, which preserved 83–97% of sequences as non-chimeric. ASVs were classified using the classify-sklearn function and the gg-13-8-99-515-806-nb-classifier.qza reference. Phylogenetic analysis was performed using the align-to-tree-mafft-fasttree function, and weighted and unweighted unifrac distances were calculated using core-metrics-phylogenetic. Data was imported into R for further analysis and visualization using the qiime2R [21], phyloseq [23] and ggplot2 [24] packages. Prevalence was calculated at the family level and was measured as the ratio of samples containing the family. Sparsely distributed ASVs were eliminated prior to diversity analysis by screening samples to only include ASVs that were observed at least 4 times in two or more samples, reducing the total number of observed ASVs from 113,232 to 40,178.

## Results

### SARS-CoV-2 probe testing on the LLMDA array

**Fig 1:**
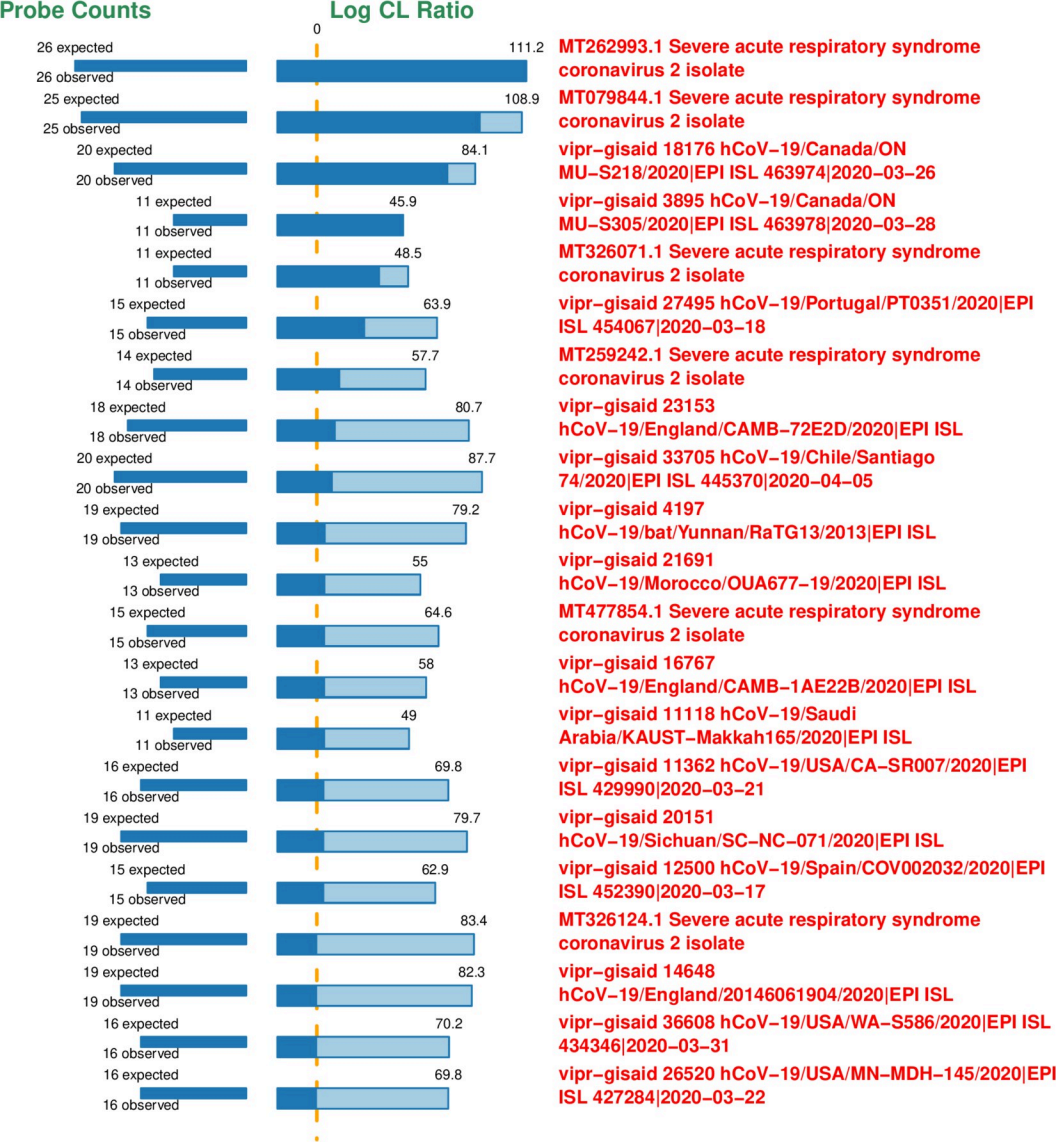
LLMDA result of synthetic SARS-CoV-2 RNA control (Twist Bioscience). The array was analyzed using the 99% threshold of signal above random controls. The light and dark colored portions of the bars represent the unconditional and conditional log-odds scores, respectively. The conditional log-odds scores show the contribution from a target that cannot be explained by another, more likely target above it. The unconditional score illustrates that some very similar targets share a number of probes.

The LLMDA successfully detected all positive control DNAs tested. Fig 1 is an example array result using the synthetic SARS-CoV-2 RNA control (MT007544.1) (Twist Bioscience). The outputs result includes log-odds ratios and the detected versus expected array features. The light and dark colored portions of the bars represent the unconditional and conditional log-odds scores, respectively. In this experiment, the target genome on the array with the closest match to the experimental sample is MT262993.1. The next closest is MT079844.1. The scores of these two closest matched sequences are very similar and correspond well to the identity of the SARS-CoV-2 control.

### LLMDA analysis of all samples

The updated LLMDA detected 358 unique species across all 203 samples. An example of an LLMDA results summary is shown in Table 1. This is from a SARS-CoV-2 positive sample (#217). For simplicity, only the ten most frequently detected species are shown in this table. The columns show the iteration of analysis, conditional scores, the number of probes expected, the number of probes detected, and the family, species and the genomic sequence level detection. In this sample, several species from the *Streptococcaceae*, *Prevotellaceae*, and *Veillonellaceae* families were detected by the LLMDA, along with SARS-CoV-2 from *Coronaviridae*. The entirety of targets identified by the LLMDA from all samples are compiled in S2 Table.

**Table 1:** LLMDA results summary example.

Iter sel	Condi-tional score	Probes expected	Probes detected	Family	Species	Target description
**0**	210	48	40	Streptococcaceae	Streptococcus pneumoniae	NC_014498.1 Streptococcus pneumoniae 670-6B, complete genome
**1**	207	27	26	Prevotellaceae	Prevotella sp. P5-108	1 Prevotella sp. F0091 Scaffold
**2**	111	26	26	Coronaviridae	Severe acute respiratory syndrome-related coronavirus	MT262993.1 Severe acute respiratory syndrome coronavirus 2 isolate SARS-Cov-2/human/PAK/Manga1/2020, complete genome
**3**	110	25	23	Prevotellaceae	Prevotella oris	1 Prevotella veroralis DSM 19559 = JCM 6290 strain DSM 19559 D464DRAFT_scaffold000
**4**	87	25	25	Coronaviridae	Severe acute respiratory syndrome-related coronavirus	MT079844.1 Severe acute respiratory syndrome coronavirus 2 isolate SARS-CoV-2/human/CHN/WHUHnCoV002/2020, complete genome
**5**	85.9	23	18	Streptococcaceae	Streptococcus gallolyticus	NZ_CP018822.1 Streptococcus gallolyticus subsp. gallolyticus DSM 16831, complete genome
**6**	75.5	15	14	Veillonellaceae	Veillonellaceae bacterium SB90	1 Veillonella sp. AF42-16 AF42-16.Scaf
**7**	69.8	20	20	Coronaviridae	Severe acute respiratory syndrome-related coronavirus	vipr-gisaid_18176 hCoV-19/Canada/ON_MU-S218/2020|EPI_ISL_463974|2020-03-26
**8**	67.6	28	23	Streptococcaceae	Streptococcus pneumoniae	1 Streptococcus pneumoniae strain SMRU693, whole genome shotgun sequence
**9**	63.3	19	19	Prevotellaceae	Prevotella timonensis	1 Prevotella nanceiensis DSM 19126 = JCM 15639, whole genome shotgun sequence
**10**	52.5	19	16	Prevotellaceae	Prevotella marseillensis	1 Prevotella pallens strain DSM 18710 Ga0131133_1

LLMDA results summary example from SARS-CoV-2 positive sample #217 of the CDPH sample set. Only the ten most frequently detected species are shown here.

**Fig 2:**
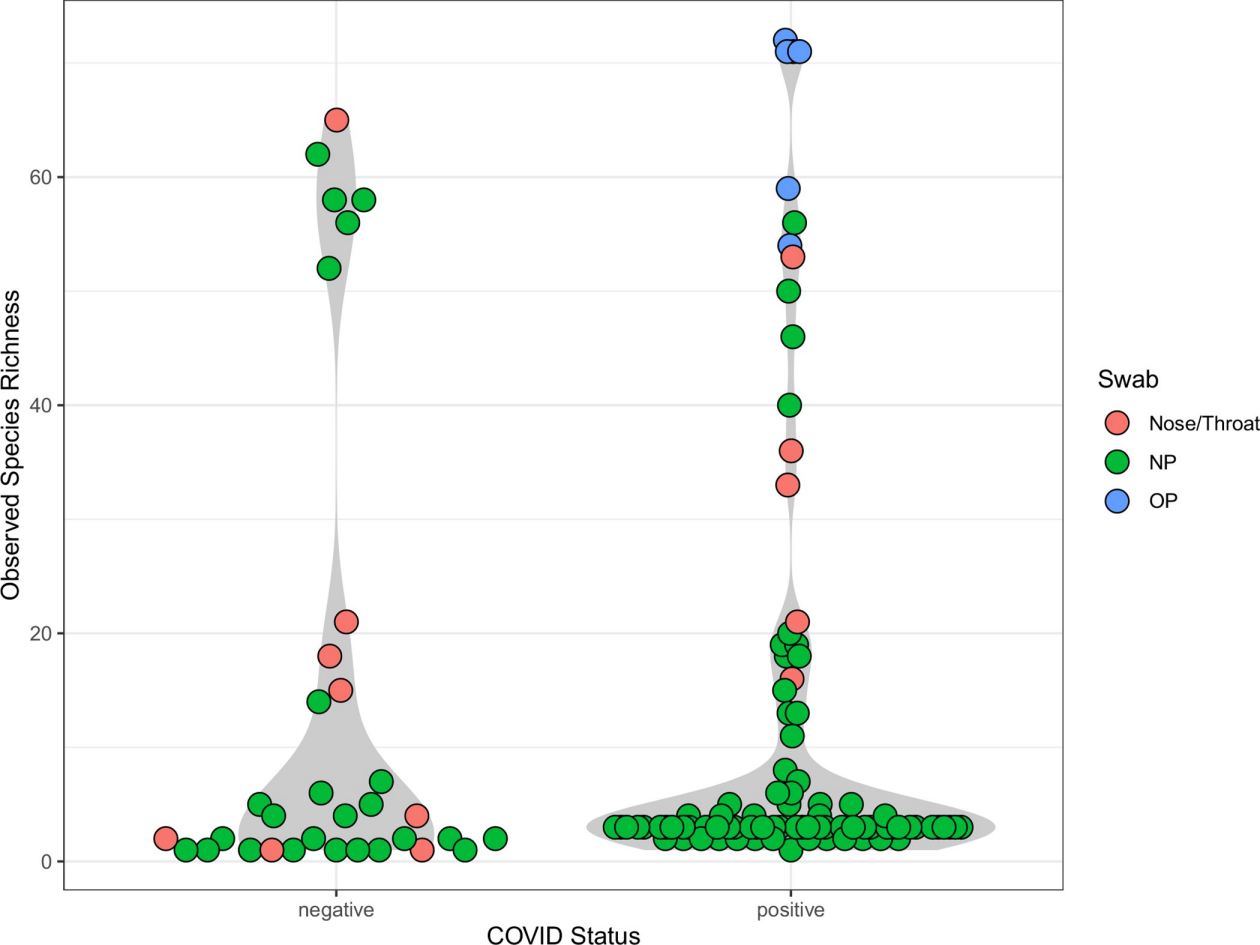
Observed species richness in SARS-CoV-2 positive vs negative samples detected by the LLMDA. Samples with no species detected are not included. The samples were coded by color based on their types: nose/throat swabs are shown in red circles; NP swabs are shown in green circles; OP swabs are shown in blue circles.

**Fig 3:**
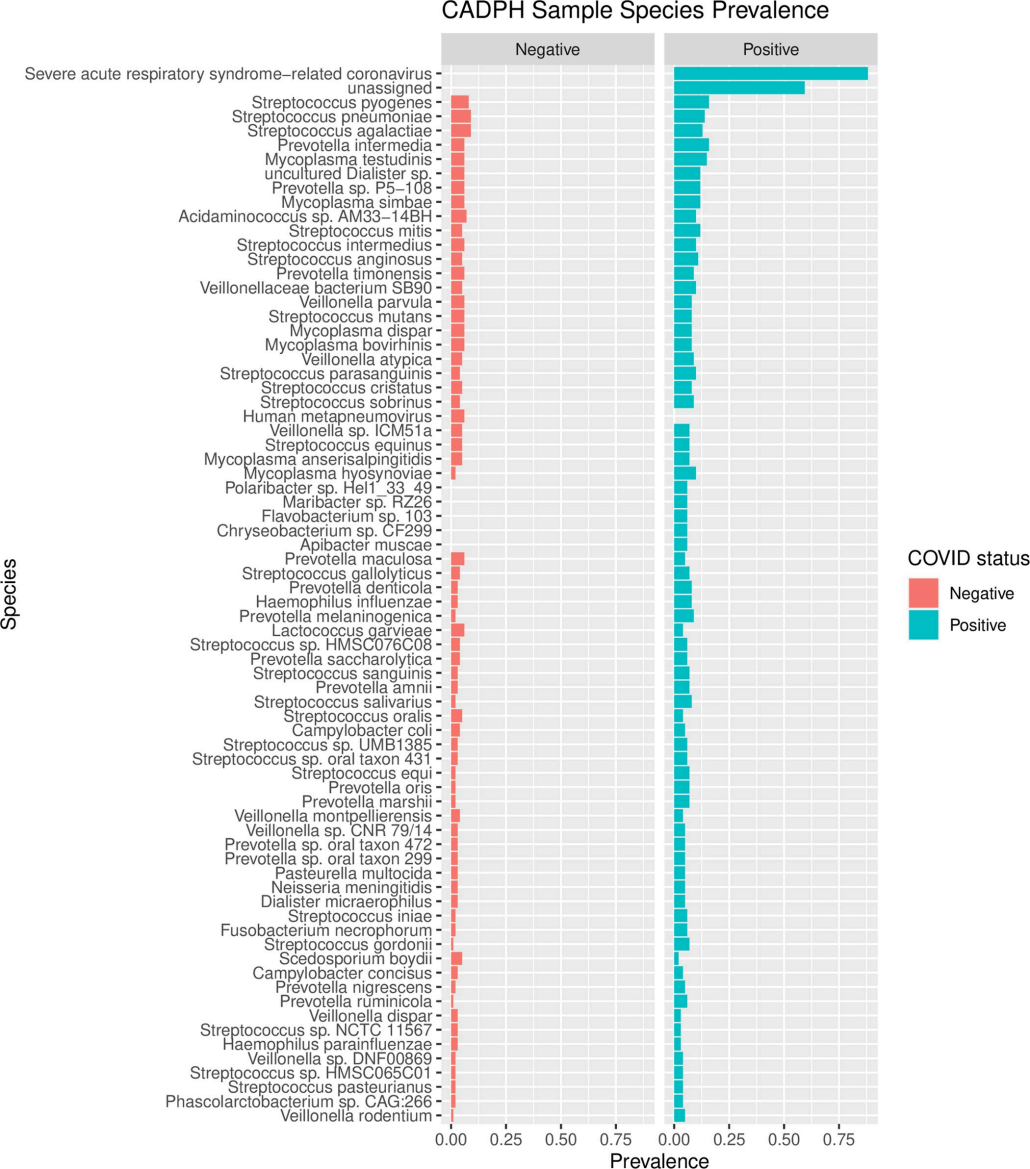
The prevalence of species detected in SARS-CoV-2 positive and negative samples using the LLMDA. Prevalence is measured as the fraction of samples in which the taxon was found. Species with a prevalence less than 5% across all samples are not shown.

Overall, viral and bacterial taxa were detected from 125 samples (62%), 92 SARS-CoV-2 positive samples and 33 SARS-CoV-2 negative samples. There was no significant difference (p = 0.1994) in the number of species detected in SARS-CoV-2 positive and negative samples (Fig 2). The OP samples were more diverse than the NP (P = 1.002e-6) and Nose/Throat (P = 9.938e-5) samples. The microbial diversity of NP and Nose/Throat samples were not significantly different (P = 0.071). The species that were detected in at least 5% of the samples are shown in Fig 3. The prevalence is calculated using the number of samples in which a species was detected vs all samples tested. SARS-CoV-2 was the only species that displayed a significant difference (adj P = 1.207e-27) in prevalence between SARS-CoV-2 positive and negative samples with all other detected species having an adjusted P-value of 1. The most prevalent bacteria detected among the 203 samples were *Streptococcus pyogenes* and *Streptococcus pneumoniae*. The family level comparison using prop.test also showed that Coronaviridae was the only family with significant difference (adj P value = 5.784e-36) with all other detected family level taxa having an adjusted P-value of 1 **(**S1 Fig**)**. The other most common families of bacteria detected included *Mycoplasmataceae*, *Streptococcaceae*, *Prevotellaceae* and *Veillonellaceae* (S1 Fig).

### SARS-CoV-2 detection: RT-qPCR vs LLMDA

The LLMDA showed 91% concordance with SARS-CoV-2 RT-qPCR, with LLMDA detecting SARS-CoV-2 from 92 of 101 SARS-CoV-2-positive samples, including all samples with a Ct <23 (Table 2). Eighty-nine samples were LLMDA positive for SARS-CoV-2 at the default 99% threshold above random controls while 3 samples were positive at 95% above random controls. The Ct values for the 3 samples detected at 95% threshold were 25.5, 30.6, and 33.2, respectively. For samples with Ct ≥ 23, the LLMDA detection rate was inconsistent with the PCR results and Ct values. For example, the LLMDA detected SARS-CoV-2 from a sample with Ct = 34.8 but failed to detected SARS-CoV-2 in samples with Ct values as low as 23, 25, and 26.

**Table 2:** SARS-CoV-2 detection results.

SARS-CoV-2 LLMDA Result	Number of samples	PCR Ct values
Detected	92^a^	13.8–34.8
Not detected	9	23–33.2

Summary of LLMDA detection compared to SARS-CoV-2 RT-qPCR Ct values.

^a^Eighty-nine samples were LLMDA positive for SARS-CoV-2 at the default 99% threshold above random controls while three samples were positive at 95% above random controls.

### Co-circulating pathogens from COVID-19 samples

The species detected by LLMDA were compared against the list of pathogens on the virulence factor database (VFDB) (http://www.mgc.ac.cn/VFs/main.htm). LLMDA identified 8 viral species in the CDPH samples (Table 3). SARS-CoV-2 was the only human viral pathogen detected in the samples listed as SARS-CoV-2 positive (92 out of 101). Ten percent (12/102) of the SARS-CoV-2 negative samples were positive for known pathogens, including human metapneumovirus (6/102) (all NP samples), Betacoronavirus 1 (1/102) (NP sample), Hepatitis B (1/102) (NP sample), Influenza B (1/102) (Nose/throat swab), and Human parvovirus B19 (1/102) (NP sample). Betacoronavirus 1 is a promiscuous CoV species that includes human coronavirus OC-43, bovine coronavirus and other coronaviruses [25].

**Table 3:** Viral species detected using the LLMDA.

	All Samples				NP Samples				OP Samples				Nose/Throat Samples			
Species	SARS-CoV-2 Positive		SARS-CoV-2 Negative		SARS-CoV-2 Positive		SARS-CoV-2 Negative		SARS-CoV-2 Positive		SARS-CoV-2 Negative		SARS-CoV-2 Positive		SARS-CoV-2 Negative	
	Count	Prevalence	Count	Prevalence	Count	Prevalence	Count	Prevalence	Count	Prevalence	Count	Prevalence	Count	Prevalence	Count	Prevalence
Betacoronavirus 1	0	0.00	1	0.03	0	0.00	1	0.04	0	0.00	0	0.00	0	0.00	0	0.00
Hepatitis B virus	0	0.00	1	0.03	0	0.00	1	0.04	0	0.00	0	0.00	0	0.00	0	0.00
Human metapneumovirus	0	0.00	6	0.18	0	0.00	6	0.24	0	0.00	0	0.00	0	0.00	0	0.00
Influenza B virus	0	0.00	1	0.03	0	0.00	0	0.00	0	0.00	0	0.00	0	0.00	1	0.13
Human parvovirus B19	0	0.00	1	0.03	0	0.00	1	0.04	0	0.00	0	0.00	0	0.00	0	0.00
Severe acute respiratory syndrome-related coronavirus	89	0.97	0	0.00	78	1.00	0	0.00	4	0.67	0	0.00	7	0.88	0	0.00
Torque teno midi virus	0	0.00	1	0.03	0	0.00	1	0.04	0	0.00	0	0.00	0	0.00	0	0.00
Torque teno virus	0	0.00	1	0.03	0	0.00	1	0.04	0	0.00	0	0.00	0	0.00	0	0.00

Viral species detected from SARS-CoV-2 positive and negative samples. Count is the number of samples. Prevalence is the percentage of samples. The results from the 99% threshold are shown.

Twenty-six species listed by the VFDB were detected among the 203 CDPH set of SARS-CoV-2 positive and negative samples (Table 4). Most of the bacterial “pathogens” that were detected are commonly isolated from human samples and are more accurately described as opportunistic pathogens that can be present as normal or transient flora [26]. The “opportunistic” species include *Escherichia coli*, *Haemophilus influenzae*, *Klebsiella pneumoniae*, *Mycoplasma hominis*, *Neisseria meningitidis*, *Staphylococcus aureus*, *Staphylococcus epidermidis*, and nine *Streptococcus* spp. The remaining bacteria detected cause a variety of illnesses in humans including digestive infections: *Campylobacter jejuni*, *Salmonella enterica*, and *Shigella flexneri*; sexually transmitted infections: *Haemophilus ducreyi* and *Neisseria gonorrhoeae*; and pneumonia: *Acinetobacter baumannii*, *Klebsiella oxytoca*, *Mycoplasma pneumoniae*, and *Pseudomonas stutzeri*. None of the bacteria were detected in a majority of the SARS-CoV-2 positive or negative samples. Three species of *Streptococci* were the most frequently detected bacteria among both SARS-CoV-2 positive and negative samples in all three sample types: *S*. *pneumonia*, *S*. *pyogenes* (Group A Strep), and *S*. *agalactiae* (Group B Strep) (Table 4).

**Table 4:** Pathogen species detected using the LLMDA.

	All Samples				NP Samples				OP Samples				Nose/Throat Samples			
Species	SARS-CoV-2 Positive		SARS-CoV-2 Negative		SARS-CoV-2 Positive		SARS-CoV-2 Negative		SARS-CoV-2 Positive		SARS-CoV-2 Negative		SARS-CoV-2 Positive		SARS-CoV-2 Negative	
	Count	Prevalence	Count	Prevalence	Count	Prevalence	Count	Prevalence	Count	Prevalence	Count	Prevalence	Count	Prevalence	Count	Prevalence
Acinetobacter baumannii	1	0.01	0	0.00	0	0.00	0	0.00	0	0.00	0	0.00	1	0.13	0	0.00
Campylobacter jejuni	3	0.03	0	0.00	1	0.01	0	0.00	2	0.33	0	0.00	0	0.00	0	0.00
Escherichia coli	1	0.01	0	0.00	1	0.01	0	0.00	0	0.00	0	0.00	0	0.00	0	0.00
Haemophilus ducreyi	2	0.02	0	0.00	0	0.00	0	0.00	2	0.33	0	0.00	0	0.00	0	0.00
Haemophilus influenzae	8	0.09	3	0.09	2	0.03	2	0.08	3	0.50	0	0.00	3	0.38	1	0.13
Klebsiella oxytoca	1	0.01	0	0.00	1	0.01	0	0.00	0	0.00	0	0.00	0	0.00	0	0.00
Klebsiella pneumoniae	1	0.01	0	0.00	1	0.01	0	0.00	0	0.00	0	0.00	0	0.00	0	0.00
Klebsiella variicola	1	0.01	0	0.00	1	0.01	0	0.00	0	0.00	0	0.00	0	0.00	0	0.00
Mycoplasma hominis	2	0.02	1	0.03	0	0.00	0	0.00	1	0.17	0	0.00	1	0.13	1	0.13
Mycoplasma pneumoniae	0	0.00	1	0.03	0	0.00	0	0.00	0	0.00	0	0.00	0	0.00	1	0.13
Neisseria gonorrhoeae	1	0.01	1	0.03	1	0.01	1	0.04	0	0.00	0	0.00	0	0.00	0	0.00
Neisseria meningitidis	5	0.05	3	0.09	2	0.03	2	0.08	1	0.17	0	0.00	2	0.25	1	0.13
Pseudomonas stutzeri	1	0.01	0	0.00	0	0.00	0	0.00	1	0.17	0	0.00	0	0.00	0	0.00
Salmonella enterica	1	0.01	0	0.00	1	0.01	0	0.00	0	0.00	0	0.00	0	0.00	0	0.00
Shigella flexneri	1	0.01	0	0.00	1	0.01	0	0.00	0	0.00	0	0.00	0	0.00	0	0.00
Staphylococcus aureus	0	0.00	4	0.12	0	0.00	2	0.08	0	0.00	0	0.00	0	0.00	2	0.25
Staphylococcus epidermidis	0	0.00	3	0.09	0	0.00	1	0.04	0	0.00	0	0.00	0	0.00	2	0.25
Streptococcus agalactiae	13	0.14	9	0.27	4	0.05	6	0.24	6	1.00	0	0.00	3	0.38	3	0.38
Streptococcus equi	7	0.08	2	0.06	1	0.01	2	0.08	6	1.00	0	0.00	0	0.00	0	0.00
Streptococcus gordonii	7	0.08	1	0.03	0	0.00	1	0.04	5	0.83	0	0.00	2	0.25	0	0.00
Streptococcus mutans	8	0.09	6	0.18	2	0.03	5	0.20	4	0.67	0	0.00	2	0.25	1	0.13
Streptococcus pneumoniae	14	0.15	9	0.27	4	0.05	7	0.28	6	1.00	0	0.00	4	0.50	2	0.25
Streptococcus pyogenes	16	0.17	8	0.24	5	0.06	6	0.24	6	1.00	0	0.00	5	0.63	2	0.25
Streptococcus sanguinis	7	0.08	3	0.09	1	0.01	3	0.12	4	0.67	0	0.00	2	0.25	0	0.00
Streptococcus suis	2	0.02	2	0.06	0	0.00	2	0.08	2	0.33	0	0.00	0	0.00	0	0.00
Streptococcus thermophilus	2	0.02	2	0.06	1	0.01	1	0.04	0	0.00	0	0.00	1	0.13	1	0.13

Pathogen species from SARS-CoV-2 positive and negative samples. Count is the number of samples. Prevalence is the percentage of samples.

### Microbial community analysis by 16S rRNA amplicon sequencing

**Fig 4:**
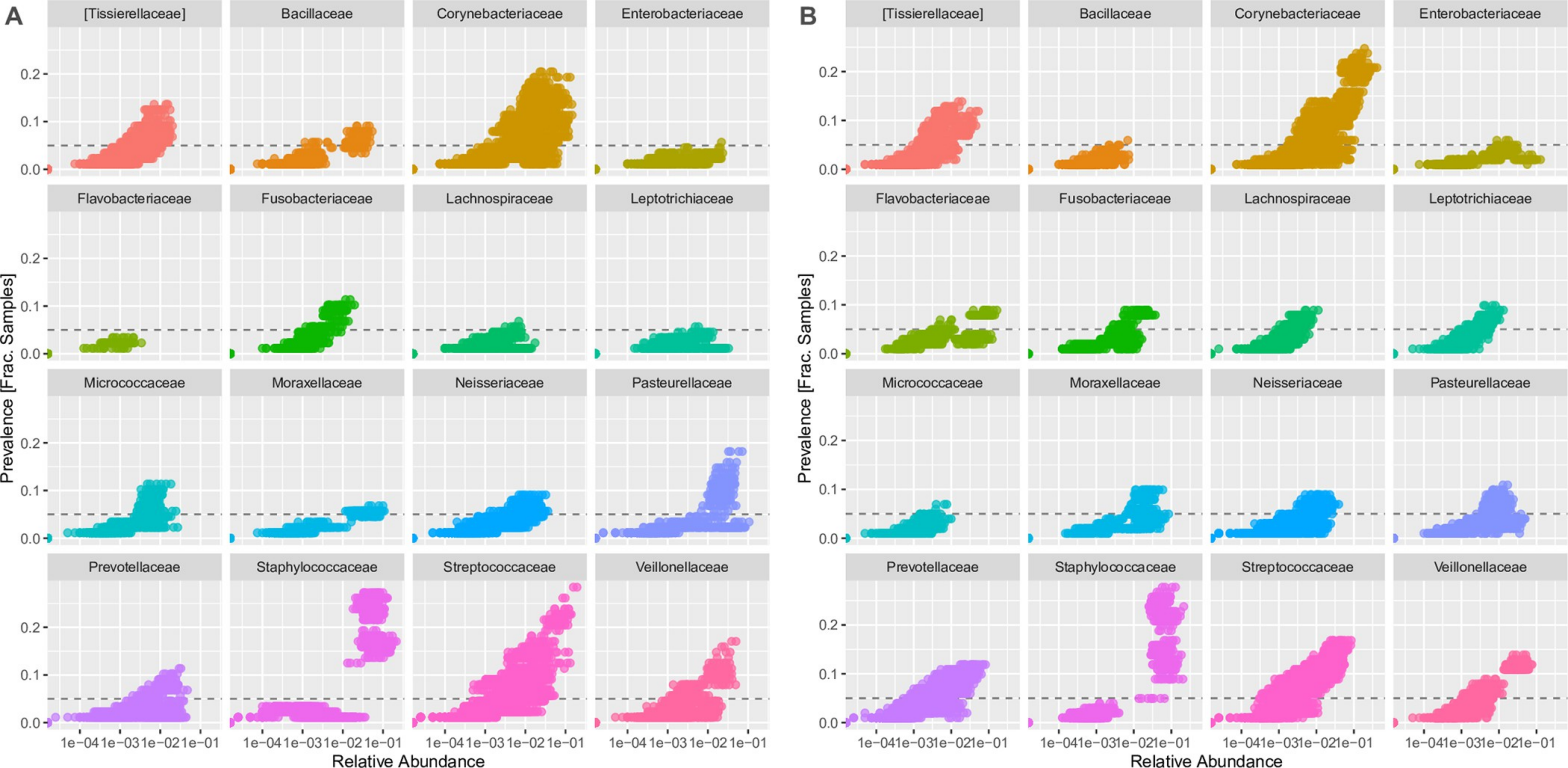
Family prevalence and relative abundance from 16S rRNA sequencing data. ASV detected in SARS-CoV-2 negative (A) and positive (B) samples. Prevalence is measured as fraction of samples in which the ASV was found. Families displayed were those with the highest overall prevalence.

**Fig 5:**
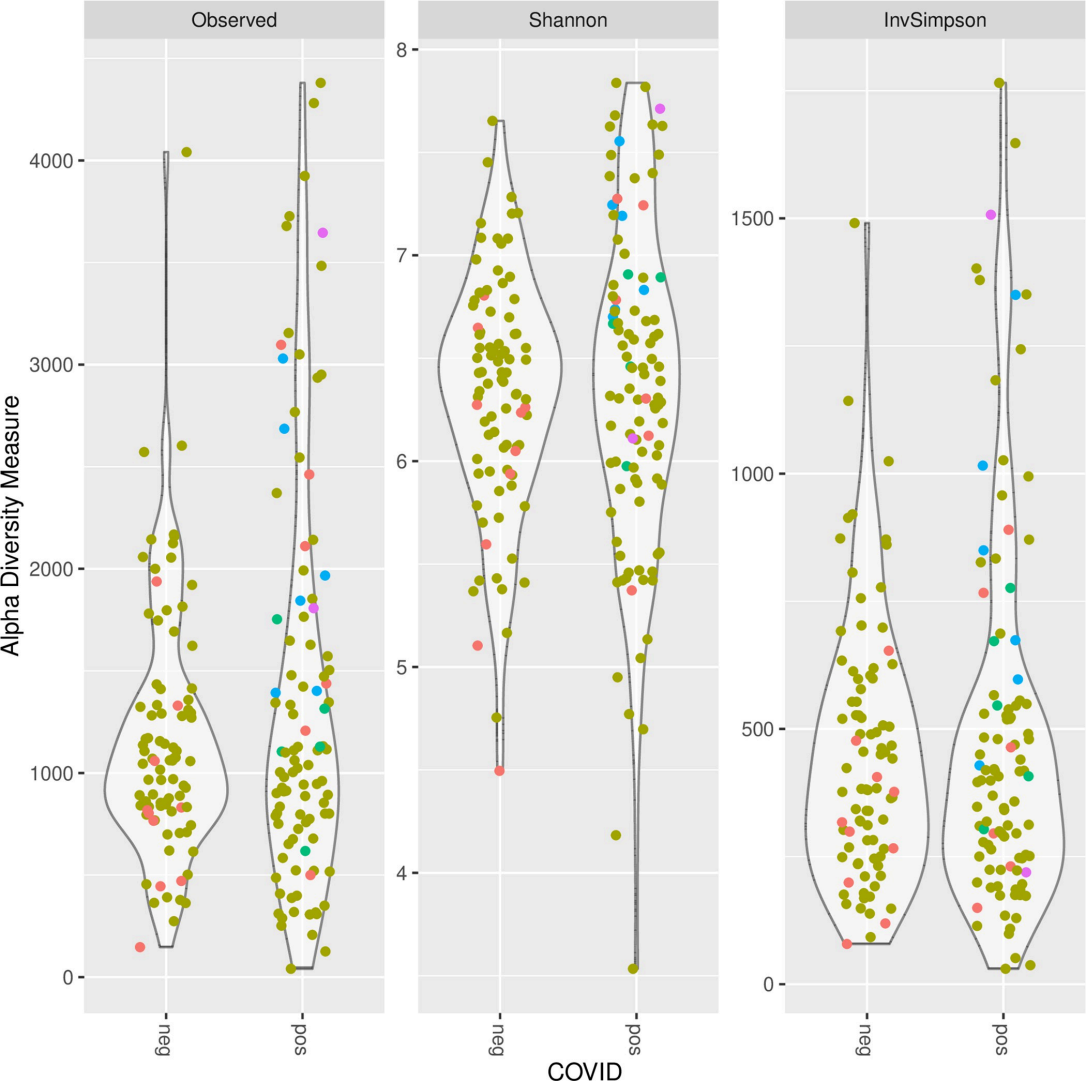
Alpha diversity analysis from 16S rRNA sequencing data. Violin plots comparing observed ASV count, Shannon index, and inverse Simpson index for SARS-CoV-2 negative and SARS-CoV-2 positive samples. P-values from Welch’s two sample t-test are displayed.

**Fig 6:**
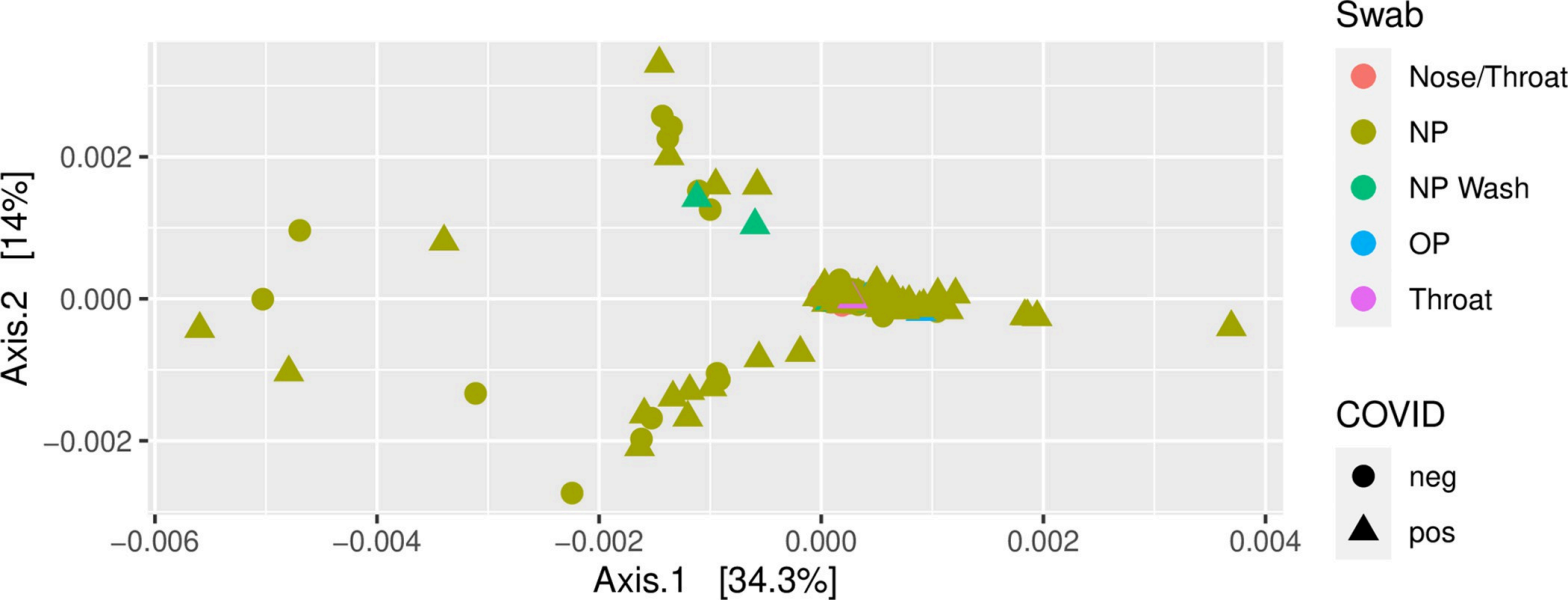
Principle component analysis of beta diversity distances (weighted Unifrac) between samples based on 16S rRNA amplicon sequencing data. Axes are the first two components representing the indicated percentages of the total variation explained. No clear separation between SARS-CoV-2 positive and SARS-CoV-2 negative samples is apparent, nor is there a significant distinction between sample types.

A 16S rRNA V4 amplicon dataset was analyzed using dada2. Most of the observed amplicon sequence variants (ASV) were very sparsely distributed, with none observed in more than 23% of the samples. An analysis of the most prevalent families shows differences between the SARS-CoV-2 positive and negative samples (Fig 4, Table 5). ASVs observed to have significantly higher prevalence among SARS-CoV-2-negative samples included bacterial families *Streptococcaceae*, *Pasturellaceae*, *Corynebacteriaceae*, *Staphylococcaceae*, *Moraxellaceae* and *Veillonellaceae*. In addition to the above families, ASVs observed to have significantly higher prevalence among SARS-CoV-2 positive samples included the *Flavobacteriaceae*, *Enterobacteriaceae*, and *Prevotellaceae* (Fig 4B). There was no significant difference in alpha diversity (Fig 5). Principal component analysis was performed on the weighted Unifrac distances between samples and the first two components plotted (Fig 6). Most samples cluster in one area of the graph. There are three other clusters that contain SARS-CoV-2 positive and negative samples and one sparsely populated area which contains only SARS-CoV-2 positive samples.

**Table 5:** 16S family level relative abundance.

Family	Total ASV^a^	ASV in NEG^b^	ASV in POS^c^	Higher in NEG^d^	Higher in POS^e^
**Prevotellaceae**	4066	1891	3694	2	34
**Corynebacteriaceae**	3368	2863	3090	76	299
**Streptococcaceae**	3100	2208	2439	117	51
**[Tissierellaceae]**	2420	2167	2038	0	1
**Veillonellaceae**	2154	1315	1691	12	42
**Neisseriaceae**	2043	1639	1810	1	1
**Lachnospiraceae**	1638	931	1405	0	0
**Leptotrichiaceae**	1268	605	1041	1	0
**Pasteurellaceae**	1137	712	1019	81	20
**Staphylococcaceae**	1083	1060	915	62	53
**Micrococcaceae**	965	720	621	0	0
**Fusobacteriaceae**	956	469	854	0	1
**Flavobacteriaceae**	904	96	849	0	330
**Moraxellaceae**	872	539	804	55	67
**Bacillaceae**	824	586	724	23	0
**Enterobacteriaceae**	772	614	495	0	45

Family relative abundance in SARS-CoV-2 negative (“NEG”) and positive (“POS”) samples from 16S rRNA amplicon analysis.

^a^Total number of ASVs assigned to the family that passed quality threshold.

^b^ASVs with prevalence > 0 in SARS-CoV-2 negative samples.

^c^ASVs with prevalence > 0 in SARS-CoV-2 positive samples.

^d^ASVs with significantly higher relative abundance (2 standard deviations) in SARS-CoV-2 negative samples.

^e^ASVs with significantly higher relative abundance in SARS-CoV-2 positive samples.

## Discussion

### Potential utility of LLMDA in co-infection analysis from pandemic diseases

Viral and bacterial co-infections could be a significant concern in treatment and management of patients during a pandemic. In previous influenza pandemics, bacterial co-infections have been a major cause of mortality. In the 2009 influenza pandemic, 1 in 4 severe or fatal cases of influenza A (H1N1) had a bacterial infection, with an apparent association with morbidity and mortality [27]. The goal of this study was to evaluate the status of co-infection in SARS-CoV-2 positive and negative samples and examine the microbiome profiles of this sample set using LLMDA and 16S rRNA sequencing technologies.

The LLMDA is a comprehensive multiplexed detection platform that includes more than 12,000 microbial and viral species. It is a tool that may be utilized for both detection and surveillance of known and emerging viral, bacterial and fungal pathogens and opportunistic pathogens. The LLMDA was recently updated to detect SARS-CoV-2, such that a single test run on LLMDA will include SARS-CoV-2 and 12,000 other microbes and viruses, providing more information than a single COVID-19 test. The LLMDA could serve as a cost-effective tool for rapid analysis of large number of samples, complementing next generation sequencing and PCR analysis. Though the turnaround time is slower than PCR, it is still faster than DNA sequencing which may take 2–3 days from sample prep to bioinformatics data analysis. The commercial version of the LLMDA, or Applied Biosystems Axiom Microbiome Array can run 96 samples at one time, with costs closer to PCR and 16S rRNA sequencing, but much lower than metagenomic sequencing [14].

### LLMDA detection of SARS-CoV-2 and co-infections from swab samples

We found that the LLMDA detected 92 of 101 (91%) of SARS-CoV-2 PCR-positive samples. The LLMDA SARS-CoV-2 discrepant (9%; 9/101) samples had PCR Ct values ranging between 23 to 33. A previous study showed that the LLMDA platform was able to detect viral RNAs in samples with Ct values of 30 or less [28]. The non-detection of SARS-CoV-2 in samples with Ct of less than 30 by the LLMDA may be related to sample quality factors, such as prolonged storage time from the original RT-qPCR test, multiple freeze-thaw cycles, and extraction method used, rather than due to a defect with the LLMDA. Indeed, the LLMDA was able to detect SARS-CoV-2 in a sample with Ct > 34.

In addition to SARS-CoV-2, the LLMDA identified other viruses and bacteria from this clinical sample set. *Streptococcus*, *Prevotella*, *Haemophilus*, *Mycoplasma*, and *Veillonella* were the most prevalent genera detected and were found in both SARS-CoV-2 positive and negative samples. At the species level, *Streptococcus pyogenes*, *Streptococcus pneumoniae*, *Streptococcus agalactiae*, *Prevotella intermedia*, *Mycoplasma testudinis* were the top five most abundant bacteria (Fig 3). Out of 203 samples, viruses and bacteria were detected from 125 samples. The other 78 samples, 9 positive for SARS-CoV-2 and 69 negative for SARS-CoV-2 were negative for viruses and bacteria. Among these 78 samples, 4 samples were nose/throat swabs (and negative for SARS-CoV-2 by PCR), the other 74 were all NP samples (9 positive for SARS-CoV-2 and 65 negative for SARS-CoV-2). These samples were collected throughout February to July of 2020 and were not from a specific month. It is likely that there was insufficient microbial or viral DNA in these samples, or the viral and bacterial concentrations were below the detection limit of the LLMDA. Previous evaluation of the 4-plex version of the LLMDA showed that the array could detect 100–1,000 copies of viral or bacterial DNA [29]. Another LLMDA study on veterinary samples correlated the sensitivity of LLMDA vs PCR and LLMDA was able to detect viruses when the Ct was less than 30 [28]. Since there were 25% (4/20) of nose/throat samples negative for any virus or bacteria, while there were 42% (74/177) of NP samples negative for any virus or bacteria, nose/throat swab seemed to be more efficient in terms of sample collection and downstream nucleic acid extraction.

The most prevalent bacteria detected in this sample set show some similarities to previous studies, though not identical. For example, in a retrospective study by Zhu et al [30], 257 laboratory-confirmed COVID-19 patients in Jiangsu Province were tested for 39 respiratory pathogens using RT-qPCR. These patients were enrolled from January 22 to February 2, 2020. Twenty-four respiratory co-infecting pathogens were identified, of which *Streptococcus pneumoniae* was the most common, followed by *Klebsiella pneumoniae* and *Haemophilus influenzae*. Lansbury et al conducted a meta-analysis of 30 studies including 3,834 patients published from January 2020 to April 2020. They found that 7% of hospitalized COVID-19 patients had a bacterial co-infection and 14% of ICU patients had bacterial co-infections with the most common bacteria identified being *Mycoplasma pneumonia*, *Pseudomonas aeruginosa* and *Haemophilus influenzae* and the most common co-infecting viruses (3%) identified as RSV and influenza A [31]. In contrast, we found *Haemophilus influenzae* was the fourth most commonly detected bacterial species (8/101 or 8%) among SARS-CoV-2 positive samples (Table 3). We did not detect influenza A or RSV from SARS-CoV-2 positive samples, consistent with decreased levels of these viruses circulating once widespread shelter-in place orders and mandated masking policies were enacted. We found that human metapneumovirus was the most prevalent virus detected in SARS-CoV-2 negative samples (Table 4). These 6 samples with human metapneumovirus detected were all collected in March or April 2020. No human metapneumovirus was detected in any of the samples collected between May to July of 2020.

Results from this study showed that only a small proportion of SARS-CoV-2 positive samples had co-detection of a viral or bacterial pathogen indicative of co-infection, consistent with other studies evaluating SARS-CoV-2 co-detections [31, 32].

### Microbiome analysis from 16S rRNA sequencing

In addition to the LLMDA analysis, we conducted 16S rRNA sequence analysis to assess bacterial microbiome diversity and prevalence in SARS-CoV-2 positive and negative samples. Our16S analysis of the ASVs (which represent species-to-strain-level resolution) suggests that the nasal microbiome is highly individualized, but there is a more common composition when phylogenetic relatedness is taken into account. ASVs within the *Streptococcaceae* and *Pasturellaceae* have a lower prevalence and/or abundance in the SARS-CoV-2 positive samples, while ASVs in the *Corynebacteriaceae* and *Moraxellaceae* have a higher prevalence and/or abundance in the SARS-CoV-2 positive samples. These results showed a similar trend to a recent study of 40 SARS-CoV-2 positive samples where the microbiota of the nasopharynx was not different in patients positive for SARS-CoV-2 RNA compared to the microbiota of patients negative for SARS-CoV-2 RNA [33]. Five phyla, namely *Firmicutes*, *Bacteroidetes*, *Proteobacteria*, *Actinobacteria*, and *Fusobacteria* comprised 98% of the sequences detected by 16S rRNA sequence analysis [33]. Another recent study showed that there was no apparent effect of COVID-19 on the nasopharyngeal microbial profiles among 33 subjects, rather, inter-personal differences were the main reason for differences in microbial composition based on the 16S rRNA sequences, regardless of COVID-19 status [34]. These observations are different from a study by Mostafa, et al. where a decrease of nasopharyngeal microbiome diversity was observed in COVID-19 confirmed patients [32]. A study of 56 SARS-CoV-2 positive and 18 SARS-CoV-2 negative patients, revealed 62 Operational Taxonomic Unit (OTU)s, mostly members of *Bacteroidota* and *Firmicutes*, that were only detected in SARS-CoV-2 positive samples, with *Prevotella*, *a genus in Bacteroidia*, found to be significantly more abundant in patients with more severe COVID-19 [35]. Therefore, though some studies have shown that COVID-19 infection causes changes of the gut microbiome [36], there is insufficient evidence that SARS-CoV-2 infection (as measured by detection of SARS-CoV-2) has a strong effect on the overall diversity of the nasal and oral microbiome. More microbiome data, in particular longitudinal studies following patients through infection and clearance, would provide clearer answers as to which populations in the nasal microbial community correlate to COVID-19 disease and related health outcomes in the patient.

### Study limitations

This was a retrospective study using residual previously tested and frozen samples. It is likely that some of the samples may have degraded over time and from multiple freeze-thaws, which may have affected the sensitivity of detection by LLMDA. Thus, some of the samples that tested negative for all viruses and bacterial could be false negatives. There were 4 samples (3 SARS-CoV-2 negative, and 1 SARS-CoV-2 positive) in this study collected from individuals who reported being asymptomatic at the time of collection, but we have no information about subsequent symptom development. The original testing done for this sample set was for SARS-CoV-2 only and no testing was pursued for other respiratory pathogens at that time.

Other potential confounding factors that may have affected the outcome of the microbiome survey include the methods employed. We used the LLMDA and 16S rRNA sequencing to detect viruses and microbes present in this set of 203 samples to characterize and analyze the microbial communities present and identify possible co-infections. We did not use PCRs targeting specific respiratory pathogens or metagenomic sequencing. When compared with 16S rRNA sequencing, LLMDA is more specific, more comprehensive, but less sensitive. LLMDA uses random amplification while 16S rRNA sequencing uses targeted amplification of the 16S rRNA region to enrich for 16S rRNA gene region. Neither the LLMDA nor the 16S rRNA sequence analysis showed significant differences in the microbiome diversity between SARS-CoV-2 positive and negative samples.

## Conclusions and recommendations

In summary, we conducted a study using the LLMDA and 16S rRNA sequencing to evaluate co-infecting pathogens and the microbiome from SARS-CoV-2 positive and negative oral, nasal or nasopharyngeal swab samples collected between February and July, 2020. We found that from the 203 samples, 62% of samples were positive for one or more viruses and/or bacteria. Beyond SARS-CoV-2, the most prevalent detected pathogens were the bacterial species *Streptococcus pyogenes* and *Streptococcus pneumoniae*. There was no significant difference in the number of additional species detected from SARS-CoV-2 positive vs negative samples. The samples collected overlapped with the start of the quarantine in most Northern California counties. It is possible that transmission of other co-circulating pathogens such as influenza was reduced due to the quarantine. The clinical data associated with the samples collected were limited, therefore the presence of co-infections cannot be correlated with clinical symptoms. Future studies using samples with well-characterized clinical data will further elucidate the possible roles that the microbiome and co-infections play in COVID-19 infection, disease progression, and mortality.

## Supporting information

S1 Fig The prevalence of families detected in COVID-19 positive and negative samples using the LLMDA. Prevalence is measured as the fraction of sample in which the taxon was found. Species with a prevalence less than 5% across all samples are not shown.
(PDF)

S1 Table List of all samples run on the LLMDA. The table includes swab type, date of sample collection, SARS-CoV-2 positive or negative by PCR, and microarray detection (if SARS-CoV-2 and microbiome were detected).
(XLSX)

S2 Table LLMDA targets detected metadata. This table includes all the target sequences detected from the 125 samples.
(TXT)
