# Supplementary figures and images for: Evaluation of co-circulating pathogens and microbiome from COVID-19 infections
Source: PLoS One. 2022 Dec 1;17(12):e0278543. doi: 10.1371/journal.pone.0278543 (PMC9714956; doi:10.1371/journal.pone.0278543)

# CADPH Sample Family Prevalence

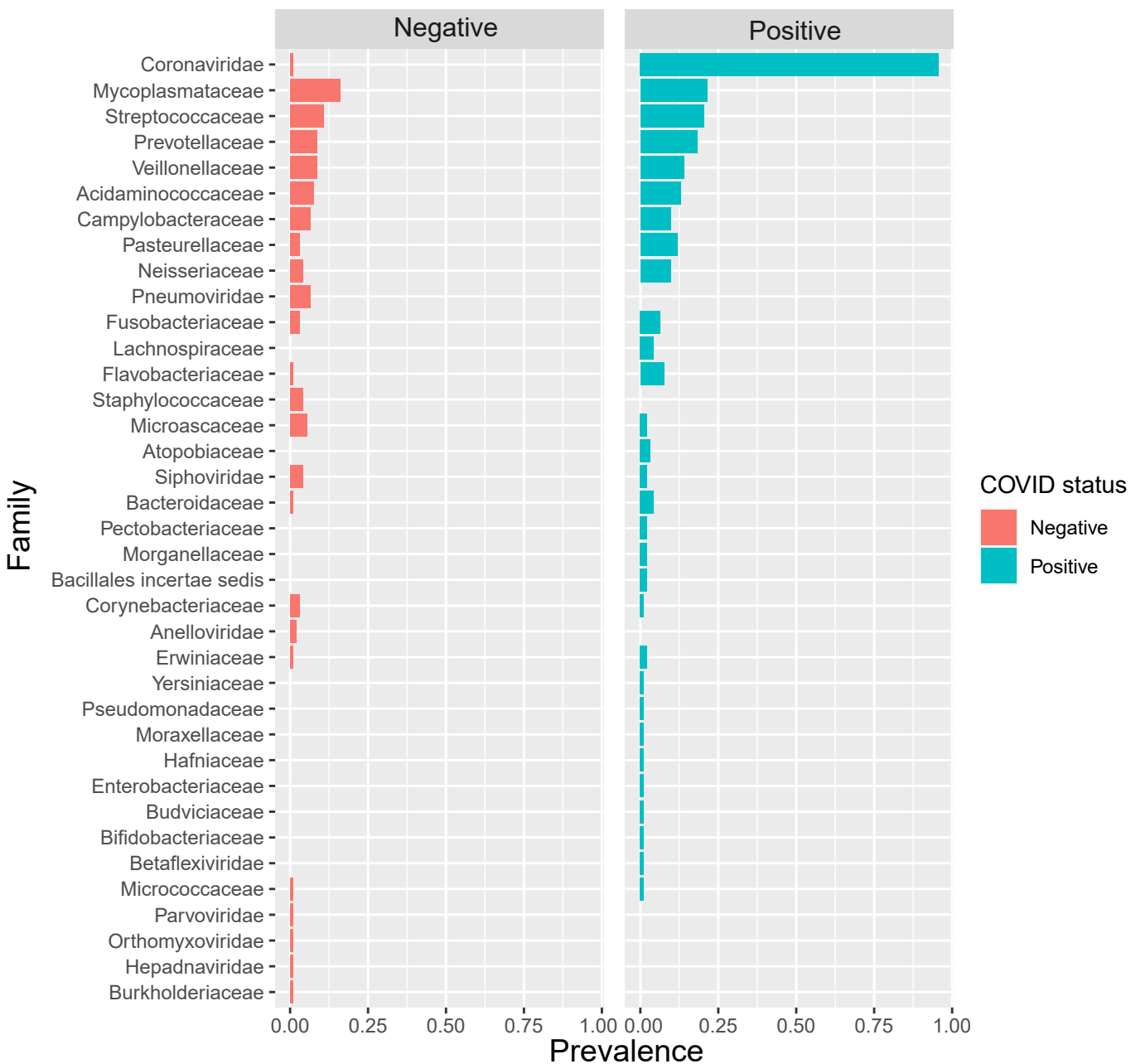

Supplement: S1 Fig — Prevalence is measured as the fraction of sample in which the taxon was found. Species with a prevalence less than 5% across all samples are not shown. (PDF) [file pone.0278543.s001.pdf]
